# Molecules produced by probiotics prevent enteric colibacillosis in pigs

**DOI:** 10.1186/s12917-017-1246-6

**Published:** 2017-11-15

**Authors:** Ricardo Nordeste, Akalate Tessema, Sapana Sharma, Zlatko Kovač, Chuan Wang, Rocio Morales, Mansel William Griffiths

**Affiliations:** 1 MicroSintesis Inc., Duffy Research Centre, NRC Building 28, 550 University Ave, Charlottetown, PE Canada; 2 Canadian Research Institute for Food Safety, University of Guelph, Guelph, ON N1G 2W1 Canada; 3 Department of Veterinary Clinical and Animal Sciences, Faculty of Health and Medical Sciences, University of Copenhagen, Dyrlægevej 16, 1870, Copenhagen, Frederiksberg C Denmark

**Keywords:** *E. coli*, Probiotics, Bioactive molecules, Proteobiotics, Pigs, Enteric colibacillosis, Anti-virulence

## Abstract

**Background:**

With the advent of antimicrobial resistance in animal pathogens, novel methods to combat infectious diseases are being sought. Among these, probiotics have been proposed as a means of promoting animal health but problems with their use has been reported. Research has demonstrated that bioactive molecules produced during the growth of certain probiotics interfere with bacterial cell-to-cell communication, which consequently results in an attenuation of virulence in a number of pathogens, including *E. coli*. The objective of this study was to determine the efficacy of the bioactive molecules, termed proteobiotics, produced by *Lactobacillus acidophilus* in preventing enterotoxigenic *E, coli* (ETEC) infection in pigs, which is the etiological agent for enteric colibacillosis, a common disease of nursing and young pigs.

**Results:**

To achieve this, piglets were fed a preparation of the bioactive at four levels: 0, 0.5×, 1.0× and 2.0× for 7 days prior to challenge with *E. coli* K88. There were 36 pigs (18 gilts and 18 barrows) per treatment, resulting in 144 piglets in total for the study. Each pen had 6 piglets (3 gilts and 3 barrows). Only piglets with no physical abnormality or conditions were used in the trial and intact male piglets and ridglings were excluded. The bioactive continued to be fed to the pigs post-challenge. Based of fecal and demeanour scores, pigs fed the low and high dose of the proteobiotic were significanlty less likely to show symptoms of illness than pigs fed no bioactive. While not being significant, the weight gain of pigs given the proteobiotics was improved. At day 4 following challenge, almost 50% of piglets that did not receive the proteobiotic were shedding ETEC in their feces, compared with about 15% of animals receiving the supplement. There was also an indication that the proteobiotics reduced colonization of the ileum by *E. coli* K88 and improved gut health.

**Conclusion:**

This study indicates that the bioactive molecules produced by *L. acidophilus* reduces incidence of enteric colibacillosis in pigs and their use on farms would help to reduce antibiotic use.

## Background

Enteric colibacillosis is a common disease of nursing and young pigs caused by enterotoxigenic strains of *Escherichia coli*. Fimbria or pili expressed by these strains of *E coli* allow them to adhere to and colonize the absorptive epithelial cells of the jejunum and ileum. The common antigenic types of pili associated with pathogenicity are K88, K99, 987P, and F41 [1]. Pathogenic strains produce enterotoxins that cause fluid and electrolytes to be secreted into the intestinal lumen, which results in diarrhea, dehydration, and acidosis [2]. Less common strains are able to produce shiga toxin Stx2e, which may result in edema [3]. Infection in neonates is commonly caused by K88 and 987P strains, whereas post-weaning colibacillosis is nearly always due to the K88 strain.

The disease has important economic implications as it results in lower growth rates and higher mortality in infected herds [4]. It has been estimated that over a one-month period, mortality due to scour can exceed 10%; accompanied by a 1 kg loss in weaning weight among surviving piglets. This loss in weight gain can result in a 10 day extension in the time to slaughter. Thus, the costs to a 500-sow unit during an outbreak lasting one month can exceed £5000 due to deaths and £2000 due to lost growth, in addition to treatment costs [5]. The control of the disease relies mainly on prevention through good hygiene, sourcing of breeding stock and promoting immunity of sows through vaccination, but when an outbreak occurs the rapid administration of antimicrobials is called for and may need to be accompanied by oral electrolyte replacement to counteract the effects of dehydration. However, many strains associated with outbreaks of colibacillosis have developed antimicrobial resistance to the drugs commonly used to combat infection [4].

Amid ever-increasing concerns about the impact of antimicrobial resistant pathogens on human and animal health, research has focused on alternative strategies to combat infectious diseases. Of these, probiotics have been extensively studied [6]. Because of potential problems associated with the delivery of whole cells [7], attention has shifted to the use of metabolites of probiotics [8]. Griffiths and colleagues were among the first research groups to investigate the efficacy of bioactive molecules isolated following the growth of probiotics against many enteric pathogens, including *E. coli* O157:H7 [9–12], *Salmonella* [12–15], *Campylobacter* [16] and *Clostridium difficile* [17]. These bioactives, termed proteobiotics, down-regulate genes involved in adhesion to and invasion of epithelial cells through interference of cell-cell communication pathways [11, 18]. The efficacy of these molecules has been demonstrated in mouse models but their application as mediators of infection in production animals needs to be assessed. Thus, the objective of this study was to determine if bioactive molecules obtained from *Lactobacillus acidophilus* could be used to alleviate infection by *E. coli* K88 in piglets. *Lactobacillus acidophilus* La-5 was chosen as previous work has shown that this organism produces bioactive molecules that are capable of down-regulating virulence genes of a variety of enteric pathogens [10, 13, 16].

## Methods

### Preparation of bioactive material

The media components were obtained from BD Difco™ (Mississauga, ON, Canada) and chemicals were obtained from either Thermo Fisher Scientific (Burlington, ON, Canada) or Sigma-Aldrich Canada (Oakville, ON, Canada). *Lactobacillus acidophilus* La-5 was obtained from the culture collection of the Canadian Research Institute for Food Safety (University of Guelph, Guelph, Canada) as a frozen stock culture that was prepared by adding 0.5 ml of a late log phase culture of the organism grown on MRS broth (BD Difco™) to 0.5 ml of sterile glycerol in a cryovial with subsequent freezing at −80 °C. The contents of the vial were defrosted at room temperature and used to inoculate a 500 ml sterile medium bottle (VWR International, Mississauga, ON, Canada) containing 250 ml of modified MRS medium (MRS2; 10 g peptone from pancreatic digest of casein, 8 g beef extract, 4 g yeast extract, 5 g sucrose, 1 ml Tween 80, 2 g dipotassium hydrogen phosphate, 0.5 g L-cysteine HCL, 2 g diamonium hydrogen citrate, 5 g sodium acetate, 0.2 g magnesium sulphate, 0.04 g manganese sulphate in 1 l distilled water); sterilized by filtration through a 0.22 μm filter (Millipore Canada, Burlington, ON, Canada). The bottle was sealed and incubated statically at 37 °C for 48 h. This culture was then used to inoculate a sterile 5 L fermentation vessel containing CDM (250 g Whey Protein Isolate ((Ergogenics Nutrition, Vancouver, BC, Canada), 25 g sucrose in 4.5 L of distilled water). The suspension was incubated statically at 37 °C for 48 h. The cells were removed by centrifugation at 12000×g for 30 min at 4 °C (Avanti J-20 XPI, Beckman Coulter, Canada). The supernatant was frozen at −80 °C and freeze dried. The freeze dried samples were stored at −80 °C and reconstituted with sterile distilled water to 1/10 of its original volume before use.

### Feed formulation

A commercial nursery pig basal diet with no medication was used to make treatment feed. A coarse crumbled Masterbatch (Table 1) was manufactured at Shur-Gain (St. Marys, ON, Canada). This Masterbatch was mixed with the test article (at rates calculated as shown in Table 2), Sipernat® (Evonik Corporation, Parsippany, NJ) to improve flow, corn and fish meal in order to make trial feed (Table 3). Treatment feed was fed from days −7 to 14 of the study. The pigs allocated to Treatment 1 did not receive any test article. The inclusion rate of the test article in the feed was according to Table 2. The test article was mixed with Sipernat® (Evonik Corporation, Parsippany, NJ) at a ratio of 60:40. For treatment 1 (Control), only Sipernat® was added to the feed. The sequence of feed mixing in the mixer was: T 1, T 2, T 3, and T 4. The mixer was cleared of physical residue after each treatment was mixed. After finishing the mixing of T 4, the mixer was thoroughly cleaned. The feed was mixed daily to ensure the stability of the bioactive fraction.

**Table 1:** Starter Feed Masterbatch formulation

Code	Ingredient Name	Amount
90,026	OAT GROATS	274.7
90,180	SKIM MILK POWDER	183.8
90,054	WHEAT SOFT	162.2
90,644	WHEY PERMEATE AGRIMAR	120
90,032	SOYMEAL HI-PRO	116.5
90,008	CORN –BK	56.3
90,242	SOYABEAN OIL BK	27.6
90,034	STAY FAT	27
90,012	DICALCIUM PHOSPHATE -BK	7.4
90,006	CALCIUM CARBONATE -BK	5.8
90,702	LYSINE (PURE)	4.3
90,058	THREONINE	3.2
39,041	SG STARTER MICRO 2.5	2.7
90,703	METHIONINE DL PURE	2.3
90,002	SALT –BK	1.5
88,572	VITAMIN B FSP	1.3
98525A	BIOTIN 200 MG/KG FSP	1.1
98530A	VIT E 50000 FSP	0.9
90,049	LIQUID CHOLINE CHLORIDE	0.7
90,098	SIG HOG KRAVE FLAVOUR	0.5
90,308	SANTOQUIN (MIXTURE 6) 25	0.2

**Table 2:** Dose calculations for proteobiotics to be delivered per treatment

Item	D-7 to −3 (5 days)	D-2 to 4 (7 days)	D5 to 14 (10 days)
BW (kg)/pig^a^	5.4	6.6	9.2
Average daily feed intake (g)/pig	180	350	500
Daily dose in ml of concentrate (0.5 ml/kg BW of pig)	2.7	3.3	4.6
Daily dose (ml test article/kg feed)			
Treatment 1 (0×)	0	0	0
Treatment 2 (0.5×))(0.5×)	7.5	4.71	4.6
Treatment 3 (1×)	15	9.43	9.2
Treatment 4 (2×)	30	18.86	18.4

^a^Estimates on body weight (BW) and feed intake are for healthy pigs

**Table 3:** Variation of feed composition throughout the trial

Trt	Peptide dose ml/kg feed	Sipernat®	Masterbatch (grams)	Corn (grams)	Fish meal 60% protein (grams)	Total (grams)
Days − 7 to − 3^a^						
T1	0	10	915.84	32.17	42.09	1000.00
T2	7.5	5	920.40	28.16	39.00	1000.00
T3	15	10	915.84	24.02	35.39	1000.00
T4	30	20	906.86	15.69	28.43	1000.00
Days − 2 to 4^b^						
T1	0	6.29	919.22	32.29	42.24	1000.00
T2	4.71	3.14	922.10	29.60	40.47	1000,00
T3	9.43	6.29	919.22	27.00	38.16	1000.00
T4	18.86	12.58	913.51	21.66	33.78	1000.00
Days 5 to 14^c^						
T1	0	6.14	919.36	32.29	42.25	1000.00
T2	4.6	3.07	922.17	29.70	40.48	1000.00
T3	9.2	6.14	919.36	27.12	38.27	1000.00
T4	18.4	12.27	913.88	21.93	33.89	1000.00

^a^The required amount of feed for each treatment is 21 kg/day

^b^The required amount of feed for each treatment is 25 kg/day

^c^The required amount of feed for each treatment is 30 kg/day

### Study design

The study was conducted as a completely randomized block design. Each treatment had 6 pens with 6 piglets per pen. All piglets were placed in the same room and pens were randomly blocked within the room as 4 pens per block. There were 6 blocks in this study. The following randomization procedures were adopted.

### Allocation of pigs to pens

Piglets (approximately 18 days +/−2 days of age upon arrival at the test facility) were obtained from commercial sow farm(s) located in Ontario, Canada and were a commercial cross breed (Newsham Genetics). There were 36 pigs (18 gilts and 18 barrows) per treatment, resulting in 144 piglets in total for the study. Each pen had 6 piglets (3 gilts and 3 barrows).

On day −7, piglets were ear-tagged and randomly assigned to pens according to their body weight. There were 4 pens in each block. Each pen had 6 piglets (3 gilts and 3 barrows). Piglets were distributed across the range of body weights (small/medium or medium/heavy). If piglets were not able to reach feed and/or water or if they had a diarrhea or demeanor score of 3 for 3 consecutive days they were removed from the study. Each pen within each block was numbered and randomly assigned to one treatment.

The size of each pen was approximately 1.2 × 1.7 m with plastic slatted flooring. Feed and water was provided ad libitum*.* The stocking density was approximately 0.33 m^2^ per piglet at the time of placement. Temperature and humidity of the facility were in accordance with piglets’ age and size throughout the study. Minimum/maximum temperatures and humidity were recorded daily. The facility including water lines was cleaned and disinfected before the arrival of piglets.

### Identification of pigs for fecal swabbing and tissue sampling

On day −7 of study, each piglet in each pen was randomly assigned a number (1 to 6). Piglet numbers 1, 2 and 3 in each pen were used for fecal swabs on days −7, −3, 0, 2, 4 and 7 of the study. If one of these pigs died, then the next pig (#4) was used for fecal swabbing. Piglet number 6 was used for intestinal tissue and content sampling on day 2 of the study (i.e. two days post-challenge).

### Study protocol

There was a 7 d acclimation period (day −7 to day 0) before the piglets were challenged with *E. coli* K88 on day 0 of the study. At this time, all piglets were orally challenged with 3 ml of an inoculum containing approximately 10^9^ CFU/ml of *E. coli* K88. The piglets were observed twice daily from the time of placement until the last day of the study. The observation periods were at least 4 h apart. Any abnormalities observed in the piglets were recorded. Any piglets that died or were euthanized during the study were weighed and necropsied to determine the possible cause(s) of death.

The drinkers and feeders were examined daily to ensure that they were clean and working properly.

Individual animal body weights were recorded on day −7, −2, 5 and 14. Feed was added to each feeder in the morning. Remaining feed in each feeder was weighed/removed before the addition of the new feed. Each scale that was used during the study, whether it be for animals or feed, was checked daily prior to the first use and after the last use, using certified check weights, to confirm accuracy.

All piglets were individually scored for fecal consistency and appearance/demeanor from day 0 to 7 of the study using the scoring system documented in Table 4. The person responsible for fecal and demeanor scoring was blinded to treatments.

**Table 4:** Criteria used to assess fecal and appearance/demeanor scores

Fecal Sco Fecal score	Description
0	No diarrhea, normal stools
1	Pasty feces
2	Liquid feces with some evidence of solids
3	Severe watery diarrhea, with or without blood
Appearance/ demeanor score	Description
0	Normal pig, good body condition, normal activity and attention
1	Pig shows mild signs of gauntness, some hollowing at the flanks, skin is duller, pig may be less active and less attentive
2	Pig shows significant signs of gauntness, skin is pale and hair is starting to become more apparent, pig shows mild signs of lethargy and less attentive to surroundings, more reluctant to move
3	Pig shows major signs of gauntness, gray pallor to skin, hair is pronounced, back may be humped up, pig shows definite signs of lethargy, and dull demeanor

On days 0, 2, 4 and 7 of the study, fecal swabs were used to take a sample from the rectum of each of the three designated piglets in each pen. Each swab was labeled with the date, trial code, and pen and pig numbers. The swabs were sent for analysis on the day of collection. Swabs were tested for presence or absence of *E. coli* K88. If a swab was positive, then it was further tested to confirm that it was *E. coli* K88.

### Identification of fecal microbiota

The fecal swabs obtained at day 5 following infection were deposited separately into a Stomacher bag, and 2 ml PBS buffer was added into each bag. After stomaching for 5 min at 200 rpm twice, the mixture in the bag was transferred to a 1.5 ml centrifuge tube, and centrifuged at 400×g for 2 min. The supernatant was separated into a new 1.5 ml centrifuge tube, and centrifuged at 16,000×g for 5 min. The supernatant was discarded and the weight of the cell pellet was measured. The DNA was extracted from the cell pellet by using a PowerFecal DNA Isolation kit (MO BIO Laboratories, Inc., Carlsbad, CA) according to the manufacturer’s instructions. The DNA samples obtained were stored at −20 °C.

### Microbiota quantification by qPCR

#### Preparation of reference strains

The reference strains were selected based on previous studies of the pig microbiota [19]. All reference strains were obtained from the Canadian Research Institute for Food Safety (CRIFS) culture collection. Culture media and conditions are described in Table 5.

**Table 5:** Reference strains and culture conditions used in the study to assess the microbiota in fecal swabs taken at day 5 following infection with *E. coli* K88

References Strains	Culture Media and Conditions
*Bacteroides fragilis* 2_1_16	FAA^a^ supplemented with 5% defribinated sheep blood. Anaerobic, 37 °C
*Bifidobacterium longum* ATCC 15707	MRS^b^ agar. Anaerobic, 37 °C
*Clostridium clostridioforme* 2_1_49FAA	FAA supplemented with 5% blood. Anaerobic, 37 °C
*Clostridium leptum* 22–5-S 16 D5 FAA	FAA supplemented with 5% blood. Anaerobic, 37 °C
*Enterobacter aerogenes*	TSA^c^. Aerobic, 37 °C
*Eubacterium limosum* 22–5-S 1 BHI	FAA supplemented with 5% blood. Anaerobic, 37 °C
*Lactobacillus acidophilus* La-5	MRS agar. Anaerobic, 37 °C

^a^FAA: Fastidious anaerobe agar

^b^MRS: de Mann-Rogosa-Sharpe agar

^c^TSA: Tryptic Soy agar

The media for the reference strains included: fastidious anaerobe agar (FAA, Neogen, Lansing, MI, USA); tryptic soy agar (TSA, Oxoid Company, Nepean, ON); deMan, Rogosa and Sharpe agar (MRS agar, BD BBL, Mississauga, ON). Defibrinated sheep blood was purchased from Fisher Scientific, Ryegate, MT. The aerobic bacteria were cultured in an air incubator and harvested after 24 h. The anaerobic bacteria were cultured in a gas jar with anaerobe container system sachets (BD BBL, Mississauga, ON), and harvested after 48 h.

After incubation colonies were removed from the plates and suspended in phosphate-buffered saline (PBS; 10 ml). Cells were harvested by centrifugation at 16,000 × g and the bacterial pellets were rinsed twice with PBS. The final bacterial pellet was suspended in 850 μl PBS, and 100 μl of the mix was used for plate count and the rest of the 750 μl mix was centrifuged for 5 min at 16,000 × g to obtain a pellet for DNA isolation. The genomic DNA was extracted using the PowerFecal DNA Isolation Kit (MoBio Laboratories, Inc., CA) following the manufacturer’s instructions; and during the extraction process, lysozyme (20 mg/ml, 37 °C, 1 h) and proteinase K were added to improve the lysis step for total DNA extraction. The DNA concentration was determined by the NanoDrop 1000 (ThermoFisher Scientific, USA) after extraction. DNA integrity was examined by electrophoresis in a 0.8% agarose gel. The DNA samples were stored at −20 °C until use.

#### Primer selection

The presence of the selected bacterial groups was determined by quantitative PCR (qPCR) with group-specific conserved 16S rRNA gene primers. To measure total bacteria, Firmicutes phylum, *Lactobacillus* spp., Bacteroides/Prevotella group, *Bifidobacterium* spp., Enterobacteriaceae group and Clostridia Cluster XIVa, seven pairs of primers as described in Table 6 were selected for targeting respective bacterial groups [20, 21]. Previous work showed that the qPCR using these primers had efficiencies of ≥95% [22].

**Table 6:** Primers used in qPCR analysis to determine the microbiota in fecal swabs taken 5 days post-infection with *E. coli* K88. The efficiency of the qPCR was ≥95% for all primers [22]

Phylum	Class/Family/Genus	Species /Group	Primer sequence	Amplicon size (bp)
Universal	All	All	F - 5′-ACTCCTACGGGAGGCAGCAGT-3′ R - 5′-GTATTACCGCGGCTGCTGGCAC-3’	175–199
Firmicutes	All	All	F - 5’-TGAAACTCAAAGGAATTGACG-3′ R - 5′-ACCATGCACCACCTGTC-3’	157
Firmicutes	Clostridia	Cluster XIVa	F - 5’-AAATGACGGTACCTGACTAA-3′ R - 5′-CTTTGAGTTTCATTCTTGCGAA-3’	438
Firmicutes	*Lactobacillus*	spp.	F - 5’-AGCAGTAGGGAATCTTCCA-3′ R - 5′-CACCGCTACACATGGAG-3’	341
Bacteroidetes	Bacteroides/Prevotella	spp.	F - 5’-GAGAGGAAGGTCCCCCAC-3′ R - 5′-CGCTACTTGGCTGGTTCAG-3’	106
Actinobacteria	*Bifidobacterium*	spp.	F - 5’-CGCGTCYGGTGTGAAAG-3′ R - 5′-CCCCACATCCAGCATCCA-3’	244
Proteobacteria	Enterobacter-iaceae	group	F - 5’-CATTGACGTTACCCGCAGAAGAAGC-3′ R - 5′-CTCTACGAGACTCAAGCTTGC-3’	195

Following optimization, these primers were used in assays where the reaction mixture (total volume 11 μl) included: 5.5 μl of 2 × SYBR® Power Green Master Mix, 0.4 μl of forward and reverse primer (10 μM), 2 μl DNA template, and 2.7 μl nuclease-free water prepared in 96-well microtitre plates sealed with a MicroAmp® Optical Adhesive Film. The amplification conditions were: one cycle at 50 °C for 2 min; one cycle of denaturation at 95 °C for 10 min; 40 cycles at 95 °C for 15 s, then 60 °C for 1 min; and a dissociation curve analysis at 95 °C for 15 s, then 60 °C for 1 min, followed by an increase to 95 °C at a 2% ramp rate and the assays were performed in a ViiA™ 7 Real-Time PCR system (Applied Biosystems, Foster City, CA).

#### Analysis of tissue samples

On day 2 (two days post-challenge) of the experiment, 24 piglets (6 from each treatment group) were euthanized and sections of the colon and ileum approximately 10 cm in length were removed and flushed with sterile PBS to remove their contents, after which they were stored at −20 °C before being delivered to the laboratory for analysis.

To process the tissues, a length of the intestine of 61 ± 16,6 mm was cut aseptically, washed in 5 mL of sterile PBS and placed in a stomacher (Seward Stomacher® 400 Circulator, Fisher Scientific) and homogenized twice at 200 rpm for 2 min. The samples were then transferred into conical tubes and centrifuged (AllegraTM 21R Centrifuge, Beckman Coulter) at 500×g for 10 min to remove large particles, and the supernatant was then centrifuged at 5000×g for 15 min. The pellet was re-suspended in 180 μL of ATL reagent from a QIAamp mini DNA kit (Qiagen Inc., Toronto, ON, Canada). After the addition of 20 μL of Proteinase K (from the QIAamp mini DNA kit), the QIAamp mini DNA kit:DNA Purification from tissues protocol was followed to obtain gDNA from the intestinal tissues [23]. The obtained gDNA samples were checked for purity using a Nanodrop 1000 Spectrophotometer (Thermo Scientific), and also for integrity, by performing agarose gel electrophoresis.

#### *Enumeration of E. coli K88 by qPCR*

Quantitative real time PCR was performed using the ViiA™ 7 PCR system (Applied Biosystems) with Power SYBR Green Master Mix (Applied Biosystems). The bacterial number of *E. coli* K88 in the colon and ileum was determined by quantitative real-time PCR targeting a 70-bp fragment of the K88 fimbrial gene [24]. The primer sequences were:

Forward: 5′- GGTTCAGTGAAAGTCAATGCATCT

Reverse: 3′- CCCCGTCCGCAGAAGTAAC.

The PCR reaction mixture had a final volume of 25 μL, consisting of 12,5 μL of the Power SYBR Green Master Mix and 4,5 μL of both the forward and the reverse primers. In total, 20 μL of reaction mixture were used with 5 μL of gDNA extracted from gut sample. The thermocycling conditions used for real time PCR were an initial denaturation step at 95 °C for 10 min, followed by the 40 cycles of amplification and quantification at 95 °C for 15 s followed by 60 °C for 20 s. To quantify *E. coli* K88, a standard curve was produced by amplification of serial dilutions of gDNA from *E. coli* K88.

#### Statistical methods

The trial data were analyzed using the MIXED and GLIMMIX Procedures of SAS 9.2 (SAS Institute, Cary, NC, USA). The model included the fixed effects of dose, sex and time and the random effect of pen nested within dose. All 2-way and 3-way interactions among the fixed effects were included in the model initially. Dose and time were retained regardless of *p*-value but other terms were excluded if not significant at α = 0.10 while preserving hierarchy. If the pen effect was non-significant it was excluded from the model. Tukey’s Honest Significant Difference (HSD) test was used for multiple comparisons.

Proc MIXED was used to analyze continuous responses with ANOVA assumptions examined using residual analyses. Residuals were tested for normality using the Shapiro-Wilk, Kolmogorov-Smirnov, Cramer-von Mises and Anderson-Darling tests. Residuals were plotted against the predicted values and variables used in the model (dose, sex, time, pen and pig) to identify outliers and unequal variances and to determine the need for transformation. Proc GLIMMIX was used for binary data to estimate Odds Ratios of treatment effects.

For repeated measures (fecal score, demeanor, weight and feed intake), the design was a split-split-plot in time. The whole-plot experimental unit was the pen. Sex within pen was the first split. Animals were then split over time (second split, repeated measures). An auto-correlation was accounted for by testing various error structures including: Autoregressive 1 (AR(1)), Heterogenous autoregressive (ARH(1)), Toeplitz and banded Toeplitz (TOEP or TOEP(t)) and the heterogenous versions of these (TOEPH and TOEPH(t)), unstructured and banded unstructured errors (UN and UN(t)), random effect of animal and no error structure. Akaike’s information criterion (AIC) was used to determine the optimal error structure. The time animals were sick (either grouped as scores of 2 or 3 or a score of 3), was a split-plot, with pen nested within dose as the whole-plot experimental unit.

The *P* values reported throughout are Adjusted P values (adjusted for multiple comparisons, giving the null hypothesis multiple chances to fail). As such, they are very conservative (higher *P* values) and, as such, it is recommended to use any adjusted *P* value <0.1 as significant.

For the microbiota data, the mean and standard deviation of the populations were calculated from standard curves of Ct against cell numbers obtained from the reference strains for total bacteria, Fircumites, *Lactobacillus* spp., Clostridial cluster XIVa, Bacteroides or Prevotella spp., *Bifidobacterium* spp., and Enterobacteriaceae. A one-way ANOVA (analysis of variance) with post-hoc Tukey HSD (Honestly Significant Difference) Test was used to calculate differences among treatments using GraphPad Prism 5 for Windows Version 5.01. A *P* value of <0.05 was considered significant.

## Results

### Fecal scores

The fecal scores observed for pigs in T 1 (control) and T 2 (0.5× dose) were significantly different (*p* = 0.004) (Table 7). When a fecal score of ≥ 2 is used as an indication that a pig is sick, the odds of a pig being sick in the control group (T 1) are 2.862 (1.359, 6.030) times higher than the odds of a pig being sick if administered the bioactive at a 0.5× dose (T 2).

**Table 7:** Effects of treatments on average fecal scores at daily intervals following infection with *E. coli* K88

Trt	Description	DO	DI	D2	D3	D4	D5	D6	D7
1	Challenged + Ox	0.06	0.39	1.16a	1.01	1.28	0.83a	0.52	0.33
2	Challenged +0.5×	0.06	0.25	0.67ab	0.77	0.67	0.17b	0.13	0.07
3	Challenged + lx	0.06	0.08	0.8 lab	1	1.23	0.63ab	0.37	0.33
4	Challenged +2×	0.03	0.25	0.56b	0.57	0.82	0.23ab	0.17	0.10
SEM		0.04	0.08	0.14	0.18	0.22	0.16	0.11	0.09
*P*-value		0.952	0.082	0.045	0.277	0.167	0.026	0.070	0.075

a-b Means within a column with no common letters are significantly different (*P* < 0.05)

There was also a significant difference (*p* = 0.029) in fecal scores among pigs in T 1 (control) and those in T 4 (2× dose). The odds of a pig being sick in the control group are 2.142 (1.066, 4.304) times higher than the odds of an animal becoming sick when fed the bioactive at a 2× dose (T 4).

A pig’s overall probability of being sick over all 8 days of the trial (day 0 to day 7 inclusive) for the control group (T 1) was 8.585% (5.546%, 13.05%); whereas the probability of a pig becoming sick in the T 2 group (0.5× dose) was 3.176% (1.828%, 5.461%) and in T 4 (2× dose) it was 4.199% (2.512%, 6.939%).

Pigs in T 3 (1× dose) were 1.381 times less likely to become ill compared to animals in the control group and their probability of becoming sick was 6.367%. This indicates that animals in this group are protected against illness but not significantly so.

### Demeanor/appearance score

Similar results were observed when the demeanor/appearance score was used as an index of infection (Table 8). Again a score of ≥ 2 was used as an indication that a pig is sick.

**Table 8:** Effects of treatments on average demeanor scores at daily intervals following infection with *E. coli* K88

Trt	Description	DO	DI	D2	D3	D4	D5	D6	D7
1	Challenged + Ox	0.06	0.06	0.17	1.07	1.40a	1.23a	0.74	0.37
2	Challenged +0.5×	0	0.03	0.06	0.70	0.77b	0.70b	0.43	0.23
3	Challenged + lx	0.03	0.06	0.19	0.97	1.17ab	1.33a	0.93	0.57
4	Challenged +2×	0	0	0.03	0.70	0.83b	0.80b	0.30	0.10
SEM		0.03	0.04	0.07	0.10	0.16	0.16	0.22	0.15
*P*-value		0.569	0.732	0.346	0.047	0.045	0.037	0.210	0.181

a-b Means within a column with no common letters are significantly different *(P <* 0.05)

In general, there was a significant effect of treatment (*p* = 0.0124) on susceptibility of pigs to infection, but a pen effect (*p* = 0.0415) was also observed when results were analysed using the Wald test. There was also the suggestion of a gender effect (*p* = 0.0809), indicating that demeanor may be affected by the sex of the animal. The odds of being sick for males was estimated to be 0.583 (0.318, 1.069) that of the odds for females being ill.

When compared to animals in T 1 (control), those administered a 0.5× dose of the bioactive (T 2) were significantly protected from illness (*p* = 0.0207), with the odds ratio for the T 1 group over T2 being 14.366 (1.414, 145.956). Indicating that animals receiving the bioactive were about 14-times less likely to exhibit severe symptoms of illness.

When compared to T 1 (control), animals in group T 4 (2× dose) were significantly less likely (*p* = 0.0831) to show signs of infection. The odds ratio (control over 2× dose) was 5.542 (0.842, 36.459). Both fecal and demeanor/appearance scores peaked at 4 days following challenge, indicating that they are good indices of infection.

### Severity and length of illness

This analysis was done by counting the number of days a pig had a fecal score of 3 before returning to a score of 1 or 0. Although there were insufficient animals in each treatment group satisfying these criteria to allow a valid statistical analysis to be carried out, trends were observed (Table 9). Far fewer pigs in T 2 (*n* = 1) and T 4 (*n* = 2) exhibited severe watery diarrhea than found in the control group T 1 (*n* = 9). The length of time that they showed this symptom was also shorter for animals in T 2 (0.03 days) and T 4 (0.1 day) than for those receiving no treatment (T1: 0.6 days). Piglets in the T 2 and T 4 group were about 3 and 2-times less likely to develop severe diarrhea, respectively, than their counterparts in the control group.

**Table 9:** Effect of bioactive on severity and length of *E. coli* K88 infection

	Treatment 1 (control)	Treatment 2 (0.5× dose)	Treatment 3 (1× dose)	Treatment 4 (2× dose)
No. of pigs with a fecal score of 3 (indicating severe diarrhea)	9	1	6	2
Average number of days exhibiting symptoms	0.5721	0.0294	0.2353	0.100
Odds ratio of untreated pigs becoming sick compared with treatment group	–	2.862	1.381	2.142

### Weight gain

Although again no significance was found due to the variability in the data, trends were apparent in the effect of treatments on the weight gained by pigs. Analysis of weight gain from day −2 (2 days prior to infection) to day 14 (end of trial, 14 days post infection; 17 days in total) revealed that animals receiving treatments 2 and 4 showed an increase in total and daily weight gain (Table 10).

**Table 10:** Median weight change in pigs over time

	Total weight change over 17 days compared to control/% change	Daily weight change (per day over 17 days) compared to control (kg)
Treatment 1 - Control		
Treatment 2–0.5×	0.633 kg/11.1%	0.037 kg
Treatment 3 - lx	−0.325 kg/−5.7%	−0.019 kg
Treatment 4 - 2×	0.807 kg/14.1%	0.047 kg

### Effect of proteobiotics on fecal microbiota

**Fig. 1:**
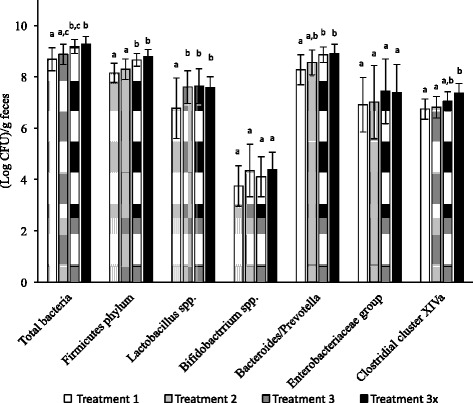
Levels of total bacteria, Firmicutes phylum, *Lactobacillus* spp., *Bifidobacterium* spp., Bacteriodes/Prevotella, Enterobacteriaeae group, and Clostridial Cluster XIVa in feces of pigs sampled at day 5 following infection. The graph shows means and standard error of the means. Different letters (*a*, *b* and *c*) indicate significantly different values (*p* < 0.05) among each treatment group. Microbiota were identified using qPCR

At the end of the study, the fecal levels of total bacteria, Firmicutes phylum, *Lactobacillus* spp., *Bifidobacterium* spp., Bacteriodes/Prevotella group, Enterobacteriaceae group, and Clostridial cluster XIVa were determined by qPCR. The population levels of each of the bacterial groups were calculated from standard curves, and the results are summarized in Fig. 1.

Compared with the control group (T1), the 0.5×-dose treatment (T2) did not significantly affect any of the bacterial groups in the microbiota except for the *Lactobacillus* group, which increased by 0.83 log CFU/g feces after this treatment. Both T3 and T4 led to an increase in population levels of most of the bacterial groups in feces except for *Bifidobacterium* spp. and the Enterobacteriaceae. The increases in counts of *Lactobacillus* in feces of pigs receiving T3 or T4 were 0.86 and 0.79 log CFU/g feces, respectively. The average increase in count of the other bacterial groups in feces of pigs subjected to T3 were 0.49, 0.51 0.31 and 0.59 log CFU/g feces for total bacteria, Firmicutes phylum, Clostridial cluster XIVa, and Bacteriodes/ Prevotella, respectively, while fecal counts in pigs receiving T4 rose by 0.59, 0.64, 0.63 and 0.63 log CFU/g feces, respectively. There were no significant differences between the fecal microbiota of pigs undergoing T3 and T4.

### Levels of *E. coli* K88 attached to ileum and colon following treatment with proteobiotic

The counts of *E. coli* K88 determined by qPCR in the ileum and colon samples and the number of positive tissue samples is shown in Table 11. Although there was not a significant difference in the number of ileum and colon samples found to be positive for the organism between treatments, there were differences in the numbers of *E. coli* present in both the ileum and colon following treatment. Lower numbers of the organism were detected in the both tissue types from pigs receiving the proteobiotic as compared to the controls.

**Table 11:** Number of *E. coli* K88 present in ileum and colon samples taken from pigs subjected to treatments and the number of tissue samples positive for the organism

Tissue	Treatment	CFU/g tissue ^a^	#animals K88 detection qPCR
Colon	T1	1.21 × 10^4^ ± 2.6 × 10^4^	5/6 ^b^
	T2	1.06 × 10^3^ ± 1.93 × 10^3^	4/6
	T3	3.42 × 10^2^ ± 3.92 × 10^2^	3/6
	T4	1.21 × 10^4^ ± 2.6 × 10^4^	2/6
Ileum	T1	1.43 × 10^8^ ± 2.02 × 10^8^	2/6
	T2	2.64 ± 0.19	2/6
	T3	2.47 × 10^8^ ± 0	1/6
	T4	0	0/6

The tissue samples were obtained from one pig (pig #6) euthanized on day 2 following infection. *E. coli* K88 was detected and enumerated using qPCR

^a^Average number of *E. coli* K88 detected in tissue samples ± standard deviation

^b^Number of tissue samples positive for *E, coli* K88/Total number of tissue samples analysed

## Discussion

Enteric colibacillosis is a common disease of nursing and weanling pigs caused by enterotoxigenic strains of *E. coli* (ETEC), which are able to colonize the small intestine. This is achieved by the fimbriae or pili of the bacteria attaching to receptors on epithelial cells of the jejunum and ileum. The common antigenic types of fimbriae associated with pathogenicity are K88, K99, 987P, and F41 [25]. Pathogenic strains produce enterotoxins that cause fluid and electrolytes to be secreted into the intestinal lumen, which results in diarrhea, dehydration, and acidosis [26]. Infection in neonates is commonly caused by K88 and 987P strains, whereas post-weaning colibacillosis is nearly always due to the K88 strain [27, 28].

With the increasing concerns surrounding the use of antibiotics in animal production, novel alternatives for maintaining animal health are needed.

Vaccination against ETEC is widely practiced on commercial pig units, and is often very effective at controlling neonatal colibacillosis. However, no single vaccine can guarantee protection against all strains [26, 29, 30]. There is also increased research effort focused on the use of biocontrol to combat enteric disease. There is evidence that bacteriophages can be used both prophylactically and therapeutically to mitigate enterotoxigenic *E. coli* infections in pigs [31] and probiotics have also been investigated for the prevention of ETEC infection either alone [26] or in combination with prebiotics (symbiotic) [32].

However, completely novel strategies to control infections that involve the use of molecules to modulate bacterial virulence are gaining credence [33]. We have coined the term proteobiotics for molecules produced by probiotics that down regulate virulence genes in both Gram positive and Gram negative bacteria [11, 13, 16, 17]. In *E. coli* it has been demonstrated that proteobiotics suppress production of quorum sensing molecules (AI2) leading to suppression of genes involved in the Type 3 Secretion System of the bacterium and the consequent alleviation of infection in a mouse model ([10, 11]. The aim of the current study was to demonstrate the efficacy of this treatment in a more relevant model: that is ETEC infection of pigs.

The proteobiotic was fed to pigs at three different dose levels calculated based on the results obtained in the mouse study [10]. The low and high dose treatments produced a significant alleviation in the symptoms of the disease; determined by measuring fecal and demeanor scores. It is interesting to note that at the 4th day following challenge almost half the pigs (44%) that did not receive the proteobiotic were shedding *E. coli* K88 in their feces; whereas only about 16% of animals receiving the low dose treatment were shedding the pathogen (data not shown). The respective percentages 7 days following infection were 6 and 0%. This suggests that not only does the treatment alleviate symptoms but it also may help prevent dissemination of the infection.

The pigs receiving the intermediate dose also showed an improvement in symptoms over the untreated animals, but the effects were not statistically significant. The reason for the lower efficacy of the intermediate dose is unclear.

It has been shown that AI-2-mediated quorum sensing is involved in K88 ETEC pathogenesis, possibly through down regulation of STa production [34] and the proteobiotic down regulates the *luxS* gene in bacteria [11, 17]. Another possible way in which the proteobiotic affects ETEC may involve interaction with host cells. When in contact with epithelial cells, genes of ETEC associated with bacterial cell signaling, including the autoinducer 2 (AI-2) synthase gene *luxS* were significantly affected [35].

Two studies have assessed the effect of a probiotic strain on the expression of ETEC virulence genes [36]. In one study, *Lactobacillus reuteri* CL9 was shown to reduce the expression of ETEC K88 heat-stable enterotoxin (ST) encoding genes (*estA* and *estB*) as well as the heat-labile toxin (LT) gene, *elt* [37]. Although these authors did not identify the mode of action, weaning piglets fed reuteran-containing diets (an exopolysaccharide produced by *Lactobacillus reuteri*) exhibited reduced the copy number of the genes encoding ST and the toxin level in the ileum, cecum and colon of piglets [38]. Pigs fed these diets containing reuteran or levan displayed a reduced average daily feed intake and a reduced average daily weight gain compared with those for pigs fed normal diets. However, feed efficiency did not differ among the diets and pigs did not develop diarrhea. It is unlikely that exopolysaccharide contributes to the results observed in the present study as previous work has identified the active fraction of the proteobiotic to contain oligopeptides [39].

For those pigs receiving the proteobiotic that did show signs of illness, the duration of the symptoms was shortened compared with the control animals, and their risk of becoming ill was between 2- to 3-times less. The fact that not all the pigs in the groups not administered the proteobiotic suffered diarrhea may be explained by their susceptibility to infection. The attachment of *E. coli* K88 to the wall of piglet intestines depends on the availability of K88 receptors, which can be found at the intestinal cell brush border and the mucus layer. Due to the existence of several different piglet phenotypes that determine the presence and type of these receptors, it can be considered that piglets generally do not uniformly respond to an *E. coli* K88 challenge. There is also a piglet phenotype that is resistant to binding of *E. coli* K88, as piglets with this phenotype do not possess the required receptors [26]. The pigs used in this study were selected to ensure they did not possess this phenotype.

Although the differences in weight gain between the pigs receiving the proteobiotic and those not was not statistically significant, the pigs that were administered the bioactive were heavier than control animals at the end of the trial, except for those pigs fed the intermediate dose. This gain in weight was not related to an increase in average daily feed intake. The average feed conversion rate for the entire length of the study was 1.256, 1.238, 1.293 and 1.236 for animals in T1, T2, T3 and T4, respectively.

Although the results indicate that feeding the proteobiotic had little effect on the gut microbiome, there were significant increases in the population of *Lactobacillus* in the feces of pigs in all treatment groups compared to the control. The ratio of *Lactobacillus* spp. to *Enterobacteria* has been used as an index of health promotion for piglets, and an increase in this ratio is related to a higher resistance to intestinal disorders [40–42]. Castillo et al. [40] found that the ratio of *Lactobacillus* spp. to Enterobacteriaceae in jejunum samples from piglets at 20 ± 2 days, determined by qPCR, was 1.29, which indicates that those weaning piglets were more resistant to enteric infections. In this study, the ratio of *Lactobacillus* spp. to Enterobacteriaceae was less than 1 for the pigs not receiving the proteobiotic, but for all the treatment groups the ratio was greater than 1, which may indicate that piglets administered the bioactive were more resistance to ETEC infections. The feeding of a mixture of probiotic *Lactobacillus acidophilus C3, Lactobacillus plantarum 1 K8 and Lactobacillus plantarum 3 K2* to pigs resulted in a significant increase in average daily feed intake, average daily weight gain and feed conversion ratio of weaned pigs within the first two weeks of administration but the effect disappeared in the following 3 to 5 weeks [43]. Giang et al. [43] also showed that administration of these probiotics resulted in a significant improvement in fecal score in a two-week period (0.22 and 0.14 for control and probiotic treatment, respectively) and a reduction in incidence of diarrhea, (measured as the sum of the total number of diarrheal piglets over the period divided by the number of piglet days in the period multiplied by 100). Thus, as well as the direct effect on virulence gene expression, the proteobiotics may act by increasing the levels of *Lactobacillus* spp. in the gut. A review of the effects of probiotics on enterotoxigenic *E. coli* infection in swine concludes that they are an attractive alternative to antibiotics [44].

The site of colonization by ETEC is the small intestine [26]. Analysis of sections of the ileum using a qPCR targeting *E. coli* K88 revealed that the average count of the organism in samples from pigs not receiving the proteobiotic was 1.4 × 10^8^ cfu/g but in sections of ileum from pigs receiving the low dose proteobiotic the average count was reduced to 2.6 cfu/g and the organism could not be detected in ileal samples taken from animals receiving the high dose. Although the number and length of ileum samples taken may not be sufficient for a valid comparison to be made, there are trends, which indicate that the administration of the bioactive reduces colonization of the small intestine by *E. coli* K88. The changes in colonization in the colon were not as apparent.

## Conclusions

This study shows that molecules, termed proteobiotics, produced by *Lactobacillus acidophilus* mitigate the effects of *E. coli* K88 infection in piglets through modulation of the pathogen’s virulence. It is anticipated that these molecules could be used as alternatives or adjuncts to reduce antibiotic use in animal production.

## Abbreviations

AI2: Autoinducer 2
ATL: Tissue lysis buffer
CDM: Chemically defined medium
ETEC: Enterotoxigenic *Escherichia coli*
gDNA: Genomic DNA
MRS: de Man, Rogosa and Sharpe
PBS: Phosphate buffered saline
